# Supplementary material for: Multidrug-Resistant Avian Pathogenic Escherichia coli Strains and Association of Their Virulence Genes in Bangladesh
Source: Microorganisms. 2020 Jul 27;8(8):1135. doi: 10.3390/microorganisms8081135 (PMC7465658; doi:10.3390/microorganisms8081135)
Supplement: Supplementary file 1 [file microorganisms-08-01135-s001.pdf]

Supplementary

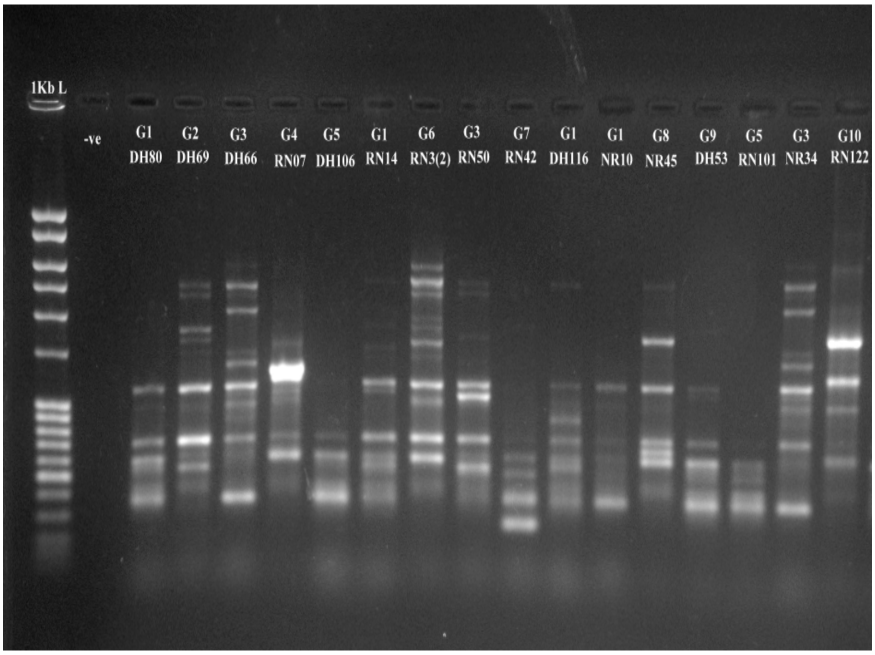

Figure S1. RAPD patterns of bacterial isolate using primer 1283. Lane 2 is negative blank control and lanes1 is molecular ladders. Lanes 3–18 are samples DH80, DH69, DH66, RN07, DH106, RN14, RN3 (2), RN50, RN42, DH116, NR10, NR45, DH53, RN101, NR34, RN122 respectively representing group 1-10.

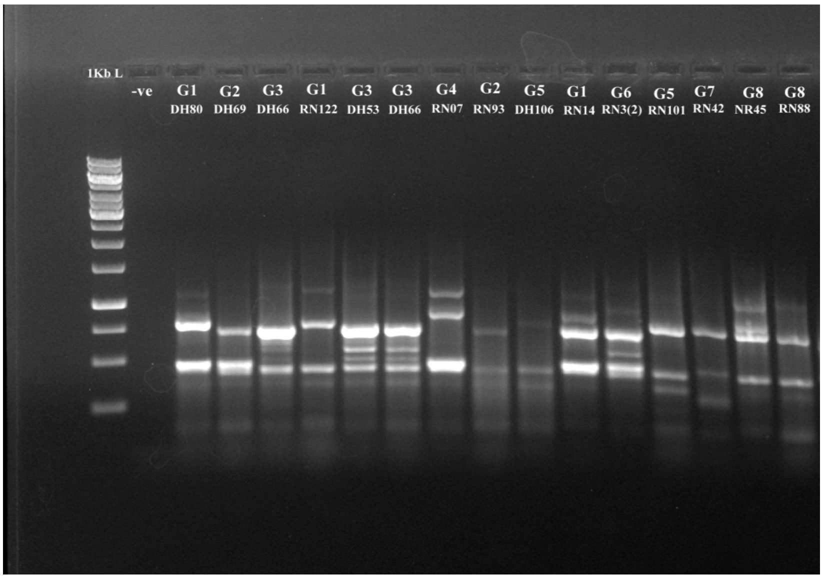

Figure S2. ERIC-PCR patterns of bacterial isolate using primer ERIC1 and ERIC2. Lane 2 is negative blank control and lanes 1 is molecular ladders. Lanes 3–17 are samples DH80, DH69,DH66, RN122, DH53, DH66, RN07, RN93, DH106, RN14, RN3 (2), RN101, RN42, NR45, RN88 respectively representing group 1-8.

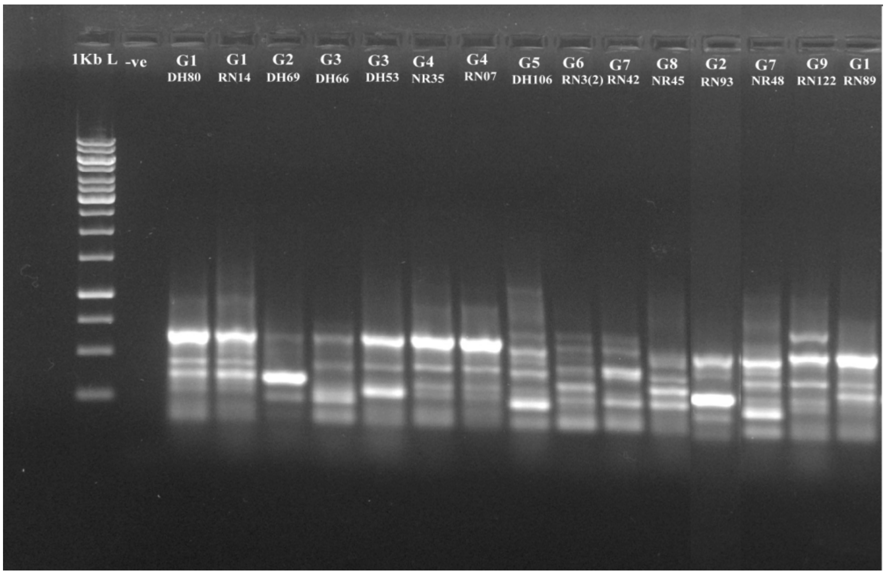

Figure S3. BOX-PCR patterns of bacterial isolate using primer BOXA1R. Lane 2 is negative blank control and lanes 1 is molecular ladders. Lanes 3–17 are samples DH80, RN14, DH69, DH66, DH3, NR35, RN07, DH106, RN3 (2), RN42, NR45, RN93, NR48, RN122, RN89 respectively representing group 1-9.

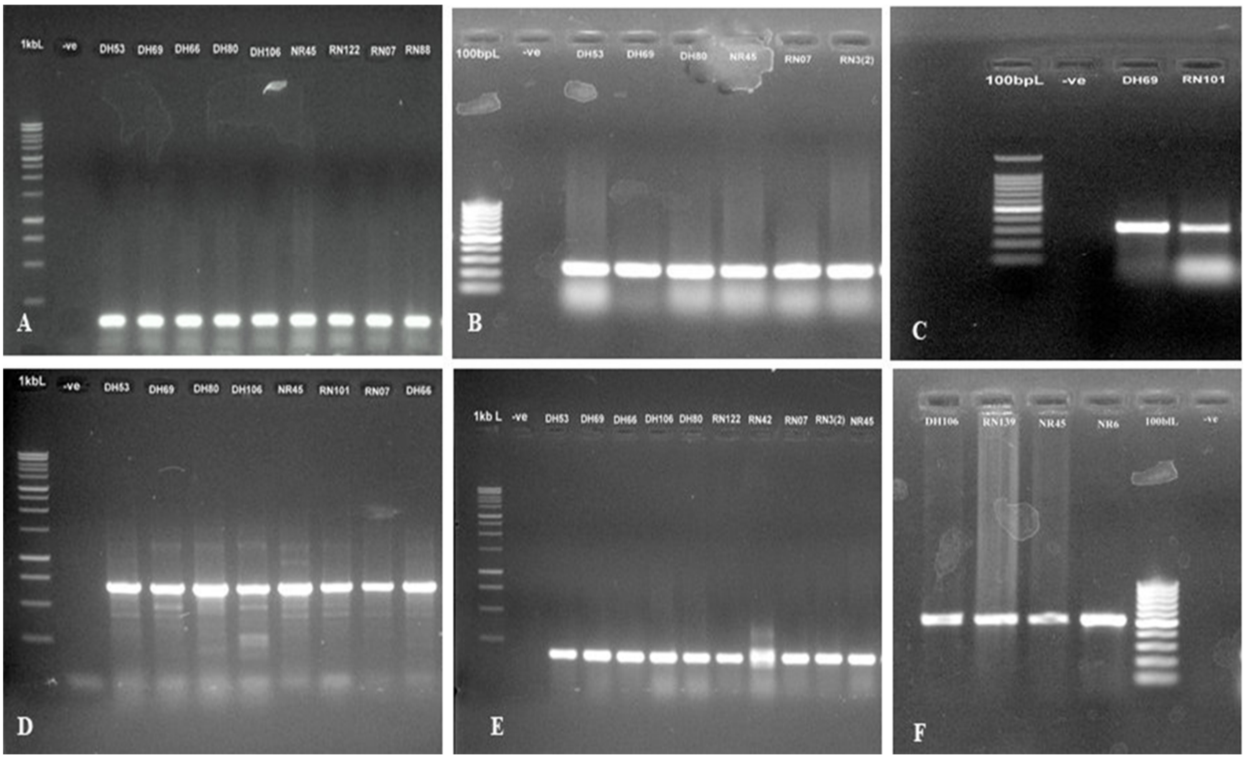

Figure S4. PCR results for the detection of *E. coli* VG among the colibacillosis cases of Bangladeshi poultry samples. A) (*uidA*: 147bp) Lane 2 is negative blank control and lanes 1 is molecular ladders (1kb). Lanes 3-11 are strain DH53, DH69, DH66, DH80, DH106, NR45, RN122, RN07, RN88. B) (*crl*: 250bp) Lane 2 is negative blank control and lanes 1 is molecular ladders (100bp). Lanes 3–10 are strain DH53, DH69, DH80, NR45, RN07, RN3 (2). C) (*papC*: 328bp) Lane 2 is negative blank control and lanes 1 is molecular ladders (100bp). Lanes 3–4 are strain DH69, RN101. D) (*ial*: 650bp) Lane 2 is negative blank control and lanes 1 is molecular ladders (1kb). Lanes 3-10 are strain DH53, DH69, DH80, DH106, NR45, RN101, RN07, DH66.E) (*fimH*: 164bp) Lane 2 is negative blank control and lanes 1 is molecular ladders (1kb). Lanes 3-12 are strain DH53, DH69, DH66, DH106, DH80, RN122, RN42, RN07, RN3 (2), NR45.F) (*cjrC*: 518bp) Lane 6 is negative blank control and lanes 5 is molecular ladders (100bp). Lanes 1-4 are strain DH106, RN83, NR45, NR6.

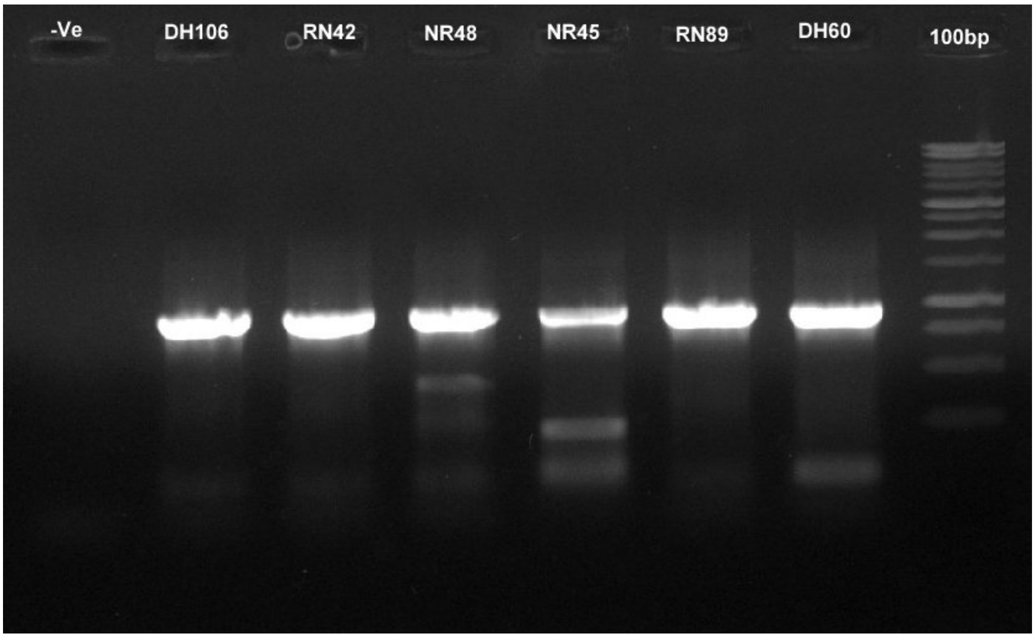

Figure S5. Representative PCR results for the detection of *Escherichia coli* phylotypes among the colibacillosis cases of Bangladeshi poultry samples. (*arp*A: 400bp), Here, Lane 8 is molecular ladders (100bp), Lane 1 is negative blank control and Lanes 2–7 are the strains DH106, RN42, NR48, NR45, RN89, DH60, respectively.

Table S1. Sequence of oligonucleotide primers of different target genes used in this study to detect pathogenic *Escherichia coli* strains.

Target gene	DNA sequence (5′→3′)	Amplified product (bp)	References
ial	GGTATGATGATGATGAGTCCA<br>GGAGGCCAACAATTATTTCC	650	[29]
bfpA	AATGGTGCTTGCGCTTGCTGC<br>GCCGCTTTATCCAACCTGGTA	334	
Stx1	CTGGATTTAATGTCGCATAGTG<br>AGAACGCCCACTGAGATCATC	150	
Stx2	GGCACTGTCTGAAACTGCTCC<br>TCGCCAGTTATCTGACATTCTG	255	
eaeA	GACCCGGCACAAGCATAAGC<br>CCACCTGCAGCAACAAGAGG	384	
lt	GGC GAC AGA TTA TAC CGT GC<br>CGG TCT CTA TAT TCC CTG TT	450	[56]
aggR	GTATACACAAAAGAAGGAAGC<br>ACAGAATCGTCAGCATCAGC	254	[57]
uidA	AAAACGGCAAGAAAAAG CAG<br>ACGCGTGGTTACAGTCTT GCG	147	[32]
cjrC	AAACCTCAGCGCAAAATCGT<br>AGGCTTCAGGAATGGGTTCA	518	[32]
fimH	GTGCCAATTCCTCTTACCGTT<br>TGGAATAATCGTACCGTTGCG	164	[29, 32]

crl	TTTCGATTGTCTGGCTGTAT<br>CTTCAGATTCAGCGTCGTC	250	[29, 32]
papC	GACGGCTGTACTGCAGGGTGTGGCG<br>ATATCCTTTCTGCAGGGATGCAATA	328	[29, 72, 75]
*hly*A	GCATCATCAAGCGTACGTTCC<br>AATGAGCCAAGCTGGTTAAGCT	534	[29]
ChuA	GACGAACCAACGGTCAGGAT<br>TGCCGCCAGTACCAAAGACA	279	
Yja A	TGAAGTGTCAGGAGACGCTG<br>ATGGAGAATGCGTTCCTCAAC	211	
TspE4C2	GAGTAATGTCGGGGCATTCA<br>CGCGCCAACAAAGTATTACG	152	[9]
*arp*A	AACGCTATTCGCCAGCTTGC<br>TCTCCCCATACCGTACGCTA	400	
arpA	GATTCCATCTTGTCAAAATATGCC<br>GAAAAGAAAAAGAATTCCCAAGAG	301	
trpA	AGTTTTATGCCCAGTGCGAG<br>TCTGCGCCGGTCACGCCC	219	

**arp*A for phylotype E and *trp*A for phylotype C

Table S2. Relative comparison among isolated Pathogenic *E.coli* from different poultry farms of three sampling locations

Farm	Sources	Isolates ID	H or D Sample	Phylotype	CRA	Pathogenic genes Number	BF	Antibiotics pattern
Farm 1	DR	DH21	H	A1	++	ND	2	ND
		DH22	H	A1	++	ND	0	ND
		DH25	D	B2(B2$_3$)	+++	*udiAcrlfim*H*ialpap*C*cjr*C	1	DoTeFCIPNAGNCS
		DH26	D	D2	+	*udiAcrlfim*H*ialpap*C	0	ND
		DH27	D	A1	+++	ND	0	ND
		DH28	D	D2	++	*udiAcrlfim*H*ialpap*C*cjr*C	0	ND
		DH52	D	B2(B2$_3$)	++	ND	0	AMPDoTeFCIPGNCS
		DH53*	D	B1	+++	*udiAcrlfim*H*ial*	4	AMPDoTeCIPNACSATM
		DH55	D	B2(B2$_3$)	++	ND	3	AMPDoFCIPNAIMPC
		DH57	D	D2	++	*udiAcrlfim*H*ialpap*C*cjr*C	0	DoTeFNAFoxGNCS
		DH59*	D	D2	+++	*udiAcrlfimHialpapCcjrC*	2	DoTeFCIPNAFoxIMPS
		DH60	D	B1	+++	*udiAcrlfim*H	0	ND
	CS	DH10	D	B2(B2$_3$)	++	*udiAcrlfim*H*ial*	3	AMPTeFNAGNNA
		DH23	D	A1	+++	*udiAcrlial*	4	ND
		DH24	H	A1	++	*udiAcrlial*	0	DoTeFNACS
		DH31	D	B2(B2$_3$)	++	ND	0	DoTeFNASATM
		DH32	H	A1	+	ND	0	AMPTeFNAFoxS
		DH33	D	A1	++	*udiAcrlial*	0	AMPDoTeFoxSATM
		DH34	D	A1	+++	*udiAcrlfim*H*ial*	0	ND
		DH35	H	A1	++	ND	0	AMPDoFCIPFoxC
		DH42	H	B1	++	ND	2	AMPDoTeCIPFoxCATM

		DH49	D	B2(B2$_3$)	+++	*udiAcrlfim*H*ial*	3	AMPTeFCIPNAFoxIMPC
		DH67	D	B2(B2$_3$)	+	ND	0	DoTeFNAS
		DH69*	D	A1	+++	*udiAcrlfim*H*ialpap*C	3	AMPDoPBCIPNAFoxIMP
	F	DH1	D	A1	++	*udiAfim*H*ialpap*C	4	DoTeFC
		DH8*	D	B2(B2$_3$)	++	*udiAcrlfim*H*ial*	3	AMPDoTeFoxIMPCATM
		DH48	D	B2(B2$_3$)	++	*udiAcrlfim*H*ial*	1	ND
		DH66*	D	B2(B2$_2$)	+++	*udiAcrlfim*H*ial*	1	AMPDoTeFCATM
	W	DH29	D	A1	++	*udiAcrlfim*H*ial*	2	FCIPNAFoxGNATM
		DH43	D	B2(B2$_3$)	+++	*udiAcrlfim*H*ial*	2	ND
		DH44	H	A1	++	*udiAcrlfim*H*ial*	0	AMPDoNAFoxIMPCATM
		DH50	D	B2(B2$_3$)	++	ND	2	TeFNACSATM
		DH51	H	D2	+	*udiAcrlfim*H*ial*	3	AMPDoTeFNAIMPC
	H	DH20#	H	A1	++	ND	1	DoTeFNAS
Farm 2	DR	DH86*	D	B2(B2$_3$)	+++	*udiAcrlfim*H*ial*	3	AMPDoTeFCIPNAFoxS
		DH87	D	B2(B2$_3$)	+++	*udiAcrlfim*H*ial*	0	TeFFoxCSATM
		DH89	D	D2	+++	*udiAcrlfim*H*ial*	1	AMPDoTeFPBNAFoxIMP
		DH90	D	B1	+++	*udiAcrlial*	0	ND
		DH93#	D	A1	+++	*udiAcrlfim*H*ialpap*C	0	AMPTePBNAFoxGNCS
		DH95	H	A1	++	ND	0	ND
		DH96	D	B2(B2$_3$)	+++	*udiAcrlfim*H*ialcjr*C	1	AMPDoFFoxCATM
		DH97	D	B2(B2$_3$)	+++	*udiAcrlfim*H*ial*	3	DoTeFoxSATM

	CS	DH73#	D	B1	+++	*udi*A*crlfim*H	0	AMPTeFCIPNAGNCATM
		DH74	D	B1	+++	*udi*A*crlfim*H	0	ND
		DH75#	D	B1	+++	*udi*A*crlfim*H*ial*	0	AMPDoTeFCIPNAGNS
	F	DH72	D	B2($B2_3$)	++	ND	0	DoTeFFoxCSATM
	W	DH71	D	D2	+	ND	0	AMPDoTeCIPNAFoxS
		DH91	H	B2($B2_3$)	+	*udi*A*crlfim*H*ial*	0	ND
		DH94	H	A1	+	*ND*	4	ND
		DH100*	D	B2($B2_3$)	+++	*udi*A*crlfim*H*ialcjr*C	1	AMPDoTeFGNS
	H	DH82	D	D2	++	*udi*A*crlfim*H*ialcjr*C	3	ND
		DH83*	D	A1	+++	*udi*A*crlfim*H	0	AMPDoTeFCIPNAFoxIMPGNS
		DH85	D	B2($B2_3$)	+++	*udi*A*crlfim*H*ialpap*C	0	ND
	IO(L)	DH76	H	B1	++	ND	0	AMPDoTeFCIPNAFox
		DH80*	D	B2($B2_2$)	+++	*udi*A*crlfim*H*ialpap*C	0	AMPDoPBNAFoxCATM
		DH103	D	B2($B2_3$)	+++	*udi*A*crlfim*H*ial*	0	ND
		DH106*	D	D2	+++	*udi*A*crlfim*H*ialpap*C*cjr*C	1	AMPDoTeCIPNAGNCS
		DH108	H	B1	++	*udi*A*crlfim*H*ial*	4	AMPDoTeNAIMPGN
		DH109	D	B2($B2_3$)	+++	*udi*A*crlfim*H*ialpap*C	0	ND
Farm 3	DR	DH152	D	B2($B2_3$)	++	*ND*	0	AMPDoTeNAGNATM
		DH162	D	A1	+++	*udi*A*pap*C*cjr*C	0	ND
		DH185	H	B1	+	ND	0	ND
		DH170	H	A1	+	ND	2	AMPDoFNAGNS
		DH116*	D	B2($B2_2$)	+++	*udi*A*crlfim*H*ial*	3	AMPDoTeFCIPNAIMPFoxGNCSATM

	W	DH141*	D	B2(B2$_2$)	+++	*udiAcrlfimHialpap*C	0	AMPDoTeCIPNAIMPC
	H	DH168	D	B2(B2$_3$)	++	*udiAcrlfimHial*	2	AMPDoTeFNAFoxATM
		DH179	H	A1	++	*udiAcrl pap*C	4	ND
	ES	DH166	D	B1	+++	*udiAcrlfimHial*	3	ND
		DH187	H	A1	+	ND	0	AMPDoFCIPNAFoxC
	IO(L)	DH144	H	B2(B2$_3$)	+	ND	0	NS
		DH145	D	B2(B2$_3$)	+++	*udiAcrlfimHialcjr*C	3	AMPDoTeFCIPNAIMPC
		DH149	D	B2(B2$_3$)	+++	*udiAcrlfimHialpap*C	4	ND
		DH173	D	B2(B2$_3$)	+++	*udiAcrlfimHial*	1	ND
		DH175	D	B2(B2$_3$)	+++	*udiAcrlfimHial*	4	AMPDoTeFCIPNAGNSATM
		DH191	D	B2(B2$_2$)	+++	ND	0	AMPTeFNAGNIMPCATM
		DH196*	H	A1	++	ND	0	AMPTeFNAS
Farm 4	DR	RN3(2)*	D	B1	+++	*udiAcrlfimHial*	1	AMPDoTeCIPGNCSATM
		RN07#	D	A1	+++	*udiAcrlfimHial*	2	AMPDoTeFNA
		RN08	H	A1	+	ND	0	ND
		RN09	H	A1	++	*udiAfimHial*	0	ND
		RN10*	D	A1	+++	*udiAcrlfimHialpapC*	1	AMPTeNAGNS
		RN14*	D	B2(B2$_2$)	++	*udiAcrlfimHial*	1	AMPDoTeFCIPIMPC
		RN22	D	B2(B2$_2$)	+++	ND	0	AMPDoTeCIPIMPC
		RN33	H	B1	+++	ND	2	ND
		RN34	D	A1	+++	*udiAfimHialcjr*C	4	AMPFNAGNCS
		RN35#	D	B2(B2$_2$)	++	ND	3	AMPDoTeFNASCATM
		RN62	D	D2	+	*udiAcrlfimHial*	0	DoFIMP
		RN63	D	B2(B2$_3$)	+++	*udiAcrlfimHialpap*C	1	ND

		RN64	D	A1	+++	*udiAcrlfimHial*	0	AMPDoTeF
		RN65	D	A1	+++	*udiAcrlfimHial*	0	ND
	CS	RN36	H	B2(B2_3)	++	*udiAcrlfimHialcjr*C	4	ND
		RN38	H	B2(B2_3)	+	ND	1	AMPDoTeCIPNAC
		RN39	D	B2(B2_3)	++	ND	0	AMPDoTeFFoxGN
		RN41#	D	B2(B2_3)	+++	*udiAcrlfimHial*	3	DoTeCS
		RN66#	D	A1	+++	ND	0	AMPDoTeCIPNAC
		RN68	H	A1	++	ND	4	ND
	F	RN42*	D	B2(B2_2)	+++	*udiAcrlfimHialcjr*C	0	AMPDoTeNA
		RN43	H	A1	++	*udiAcrlfimHial*	0	ND
	W	RN32	D	D2	+	*udiAcrlfimHialpap*C*cjr*C	1	AMPDoTeCIPNAFoxS
	H	RN16	D	A1	+++	*udiAcrlfim*H	0	AMPDoFCIPFoxATM
		RN18	D	B2(B2_3)	+++	*udiAcrlfimHialpap*C	4	AMPDoTeFFoxCS
		RN19	D	B2(B2_2)	++	*udiAcrlfimHial*	4	TeFCIPIMPCATM
		RN29	D	D2	+++	*udiAcrlfimHialpap*C*cjr*C	0	AMPDoTeNAFoxIMP
		RN30	D	B2(B2_3)	++	*udiAcrlfimHial*	2	TeFCIPFoxS
		RN31	H	B2(B2_3)	++	*udiAcrlfimHial*	0	DoTeNAFoxGN
	IO(L))	RN20	H	B2(B2_3)	+	ND	0	ND
		RN46	H	B2(B2_3)	+	ND	4	AMPDoFCATM
		RN47	H	A1	++	*udiAcrlfimHial*	0	ND
		RN48	D	B2(B2_3)	+++	*udiAcrlfimHialcjr*C	0	AMPTeFPBNAGNAT M
		RN50*	D	B2(B2_2)	+++	*udiAcrlfimHialcjr*C	0	AMPDoTeFCIP
		RN51*	D	B2(B2_3)	+++	*udiAcrlfimHial*	0	DoTeCIPNAFoxCATM
		RN52	H	B2(B2_3)	++	ND	2	ND
		RN53	H	A1	+	ND	0	ND
		RN54	D	A1	+++	*udiAialpap*C	2	AMPFPB

		RN55	D	A1	+++	*udi*A*ialpap*C	0	AMPTeCIPFoxIMPS
		RN58	D	B2(B2$_3$)	++	ND	0	AMPDoTeFCIP
		RN59*	D	D2	+++	*udi*A*crlfim*H*ialpap*C*cjr*C	2	DoTeFCIPGNCS
		RN60	D	A1	++	ND	0	ND
Farm 5	DR	RN93*	H	B1	+++	*udiAcrlfimHial*	4	AMPDoTeFNAFoxS
		RN105	D	B2(B2$_2$)	++	*udiAcrlfim*H*ialpap*C	0	ND
		RN106	H	B2(B2$_3$)	++	ND	3	DoTeCIPNAC
		RN110	D	A1	+++	*udiAcrlfimHial*	0	DoTeCIPNAS
		RN111	H	A1	++	*udiAcrlfimHial*	0	AMPDoTeNAFoxGNC
		RN112	H	A1	++	*udiAcrlfimHial*	0	AMPTeFNAFoxGN
		RN113	D	B2(B2$_2$)	+++	*udiAcrlfim*H*ialcjr*C	0	ND
		RN114	H	B2(B2$_2$)	+	ND	1	ND
		RN117*	D	B2(B2$_3$)	++	*udiAcrlfimHialcjrC*	2	AMPDoTeFNASATM
		RN118#	D	A1	+++	*udiAcrlfimHialcjr*C	0	AMPDoTeCIPNAIMPC
		RN122*	H	A1	+++	*udiAcrlfim*H	1	DoTeFC
		RN133	D	B2(B2$_3$)	+++	*udiAcrlfimHialpap*C	0	AMPDoTePBCIPGNC
		RN135	H	B2(B2$_3$)	++	ND	0	ND
		RN138	D	B2(B2$_3$)	+++	*udiAcrlfimHialcjr*C	0	AMPDoTeCIPFoxCAT M
		RN139	D	B2(B2$_3$)	++	*udiAcrlfim*H*ialcjr*C	0	DoTeFGN
	CS	RN99	H	B2(B2$_3$)	+	ND	3	ND
		RN100	D	B2(B2$_2$)	++	*udiAcrlfim*H*ialpap*C	0	ND
		RN101*	D	D2	+++	*udiAcrlfim*<u>H</u>*ialpap*C*cjr*C	1	ND
		RN102	D	D2	++	*udiAcrlfimHial*	0	ND
		RN119	H	A1	++	ND	0	AMPTeFS
	W	RN123	H	A1	++	*udiAcrlfimHial*	0	ND
		RN128	D	A1	+++	*udiAcrlfimHial*	0	ND
		RN131	D	A1	+++	*udiAcrlfimHial*	0	ND

		RN132	D	A1	++	ND	0	AMPDoFIMPS
	ES	RN83*	D	B2(B2$_3$)	++	*udiAcrlfimHial*	0	AMPDoTeFCIPFox
		RN84	D	A1	+++	*udiAcrlfimHial*	0	AMPDoFCSATM
		RN85	D	A1	+++	*udiAialpap*C	0	AMPDoTeFNAATM
		RN86	D	A1	++	ND	0	AMPDoTePBCIPFoxGN
		RN87	D	B2(B2$_3$)*	+++	*udiAcrlfimHial*	0	AMPTeC
		RN88	D	B2(B2$_3$)	+++	*udiAcrlfimHial*	1	ND
	H	RN70	D	D2	++	*udiAcrlfimHialpap*C*cjr*C	1	ND
		RN71*	D	D2	+++	*udiAcrlfimHialpap*C*cjr*C	2	AMPDoTeFCIPGNS
		RN89#	D	A1	+++	*udiAcrlfimHial*	0	DoTeFCIP
		RN90	D	B2(B2$_3$)	++	*udiAcrlfimHialpap*C*cjr*C	0	AMPFCIPFoxIMPCATM
		RN95	D	B2(B2$_3$)	++	ND	3	ND
		RN96*	D	B2(B2$_3$)	+++	*udiAcrlfimHialpap*C*cjr*C	1	AMPTeCIPCS
	IO(L)	RN97*	D	B2(B2$_3$)	+++	*udiAcrlfimHialpap*C	3	AMPDoTeCIPFox
		RN126	D	D2	++	*udiAcrlfimHialpap*C	0	ND
		RN98	D	B2(B2$_3$)	++	*udiAcrlfimHialpap*C	2	AMPDoTeFC
Farm 6	DR	NR1*	D	B2(B2$_3$)	+++	*udiAcrlfimHialcjrC*	1	AMPDoTeFNAC
		NR3	D	B2(B2$_3$)	+++	ND	0	ND
		NR4	D	B2(B2$_3$)	++	ND	0	ND
		NR6	D	D2	+++	*udiAcrlfimHialpap*C*cjr*C	0	ND
		NR9	D	B2(B2$_3$)	++	ND	1	AMPDoTeNAFoxS
		NR10#	D	B2(B2$_3$)	+++	*udiAcrlfimHialpap*C	2	AMPDoTeFPBCIPNAIMPCATM
	CS	NR13	D	B2(B2$_3$)	+	ND	0	AMPDoTeNACS
		NR14	D	A1	+++	*udiAcrlfimHial*	2	AMPDoTeFCIPNACS
		NR15	D	A1	+	ND	3	ND

		NR20	D	A1	++	*udi*A*crlfim*H*ial*	3	ND
	F	NR28	D	B2($B2_3$)	+++	*udi*A*crlfim*H*ial*	0	ND
		NR34#	D	A1	+++	*udi*A*crlfim*H*ialcjr*C	0	TeFNAFoxCS
	IO(L)	NR35	D	B2($B2_3$)	+++	*udi*A*crlfim*H*ial*	2	AMPDoTeFNAFoxIMP CS
		NR45*	D	A1	+++	*udi*A*crlfim*H*ialcjr*C	1	AMPTeNAFoxSATM
		NR46*	D	B2($B2_2$)	++	*udi*A*crlfim*H*ialcjr*C	2	AMPDoTeNAFoxIMP GNATM
		NR47	D	A1	++	*udi*A*crlfim*H*ial*	2	FCIPNACSATM
		NR48*	D	D2	+++	*udi*A*crlfim*H*ialpap*C*cjr*C	1	DoTeFNACS

DH- Dhamrai, RN- Rupganj, NR- Norshingdi, DR-Dropping, CS-Cloacal Samples, F- Feed, W-Feeding Water; H = Handler Swab; ES = Egg Surface Swab; IO(L) = Internal Organ(Liver); H-healthy, D-Diseases, BF-Biofilm Formation, CRA-Congo Red Assay, ND-Not Done; AMP = ampicillin; Te = Tetracycline; Do = doxycycline; F = nitrofurantoin; Pb = polymexin CIP=ciprofloxacin; NA=nalidixic acid; Fox=cefoxitin; IMP = imipenem;GN=gentamycin; C = chloramphenicol S3 = Sulfonamide; AZM = azithromycin; + = Positive; 0 = Not Done; 4=Not Biofilm Producer; 3 = Weak biofilm producer ; 2 = Moderate biofilm producer; 1 =Strong biofilm producer.*-Plasmid Positive; # Plasmid Negative.
